# Effects of optogenetic inhibition of a small fraction of parvalbumin-positive interneurons on the representation of sensory stimuli in mouse barrel cortex

**DOI:** 10.1038/s41598-022-24156-y

**Published:** 2022-11-12

**Authors:** Fahimeh Yeganeh, Beate Knauer, Roberta Guimarães Backhaus, Jenq-Wei Yang, Albrecht Stroh, Heiko J. Luhmann, Maik C. Stüttgen

**Affiliations:** 1 Institute of Physiology, University Medical Center of the Johannes Gutenberg University Mainz, Duesbergweg 6, 55128 Mainz, Germany; 2 Institute of Pathophysiology, University Medical Center of the Johannes Gutenberg University Mainz, Duesbergweg 6, 55128 Mainz, Germany; 3 Leibniz Institute for Resilience Research, Mainz, Germany

**Keywords:** Somatosensory system, Neuroscience

## Abstract

Inhibitory interneurons play central roles in the modulation of spontaneous network activity and in processing of neuronal information. In sensory neocortical areas, parvalbumin-positive (PV+) GABAergic interneurons control the representation and processing of peripheral sensory inputs. We studied the functional role of PV+ interneurons in the barrel cortex of anesthetized adult PVCre mice by combining extracellular multi-electrode recordings with optogenetic silencing of a small fraction of PV+ interneurons. In all cortical layers, optogenetic inhibition caused an increase in spontaneous network activity from theta to gamma frequencies. The spatio-temporal representation of sensory inputs was studied by stimulating one or two whiskers at different intervals and analyzing the resulting local field potential (LFP) and single unit (SU) response. Silencing PV+ interneurons caused an increase in LFP response to sensory stimulation and a decrease in temporal discrimination of consecutive whisker deflections. The combined effect of whisker deflection and optogenetic inhibition was highly similar to the linear sum of the individual effects of these two manipulations. SU recordings revealed that optogenetic silencing reduced stimulus detectability by increasing stimulus-evoked firing rate by a constant offset, suggesting that PV+ interneurons improve signal-to-noise ratio by reducing ongoing spiking activity, thereby sharpening the spatio-temporal representation of sensory stimuli.

## Introduction

The whisker-to-barrel cortex pathway represents a unique sensory system to study the neuronal processing of sensory information in relevant brain structures from brainstem to thalamus and finally cerebral cortex^1–3^. In the rodent primary somatosensory cortex, the whiskers are topographically represented in a chessboard-like manner, and a whisker-related cortical column has a surface area of ~ 300 µm by ~ 300 µm, containing approximately 12,000 neurons^4^. This organization of the sensory periphery in the barrel cortex offers the unique possibility to monitor the activation of a local cortical network following well-defined mechanical stimulation of a single whisker or several whiskers at defined temporal intervals. Although we have gained considerable insights into the structure and function of each cortical layer in processing simple and more complex sensory stimuli^4–6^, we do not fully understand the role of specific neuronal cell types in this process. Inhibitory GABAergic interneurons play a central role in the intracortical processing of sensory information^7^ and can be differentiated into six to ten major cell types based on morphological, electrophysiological and molecular properties^8–12^. Parvalbumin immunoreactive (PV+) neurons account for about 40% of the total population of neocortical GABAergic cells and morphologically consist of basket and axo-axonic (or chandelier) cells (for review^13^). Upon injection of depolarizing current pulses, PV+ interneurons discharge over long intervals with brief action potentials at high frequencies with little or no spike frequency adaptation (fast spiking)^14,15^. Basket cells provide perisomatic inhibition to postsynaptic excitatory neurons, both vertically within the column as well as horizontally to neighboring columns within a given cortical layer (for review^8,9^). It has been suggested that PV+ basket cells rapidly and strongly inhibit essentially every pyramidal neuron within a radius of ~ 200 µm, thereby providing an "early blanket of inhibition"^16^. However, subtypes of basket cells also fulfill other functional roles in intracortical information processing, for example, axo-axonic cells may even have an excitatory impact at the axon initial segment of the postsynaptic neuron ^9^.

Fast spiking PV+ interneurons in the cerebral cortex are involved in the generation of gamma oscillations^17^ and play pivotal roles in sensory processing (for review^18^). Optogenetic activation of fast spiking PV+ interneurons in barrel cortex suppressed stimulus-evoked firing rates while improving temporal specificity^19^. An optogenetic study in awake mice detecting naturalistic vibrissal stimuli demonstrated that "fast spiking gamma" activity improves psychophysical performance^20^. It has been further suggested that PV+ interneurons regulate the balance of excitation and inhibition (E/I) and that disturbances in this E/I balance will not only impair cortical information processing, but in prefrontal cortical circuits may even result in a range of psychiatric disorders (for review^21^).

To further study the role of PV+ interneurons in intracortical processing of peripheral sensory information, we used a combination of multi-electrode extracellular recordings and optogenetic manipulation of PV+ interneurons in the barrel cortex of anesthetized adult PVCre mouse (for review^22^). The recorded local field potentials (LFPs) provide spatiotemporal information on the synaptic activation of a local neuronal network, e.g. a cortical layer, and single unit spiking activity provides information on the suprathreshold activation of distinct cortical neurons (for review^23,24^). We expressed the light-sensitive inhibitory opsin ArchT in PV+ interneurons^25^ and transiently inhibited these neurons by laser light pulses.

Using these methods, we studied the role of PV+ interneurons in the intracortical processing of defined sensory stimulation of a single whisker or two neighboring whiskers at various inter-stimulus-intervals. We addressed the following questions: (1) What is the effect of PV+ interneuron inhibition on the temporal and spatial representation of whisker stimulation in the barrel cortex? (2) Does PV+ interneuron inhibition influence the signal-to-noise ratio (SNR) of the neuronal signal? (3) What is the impact of PV+ interneuron inhibition on the representation of single whisker stimulus sequences?

## Results

### Number of opsin-expressing neurons

**Figure 1:**
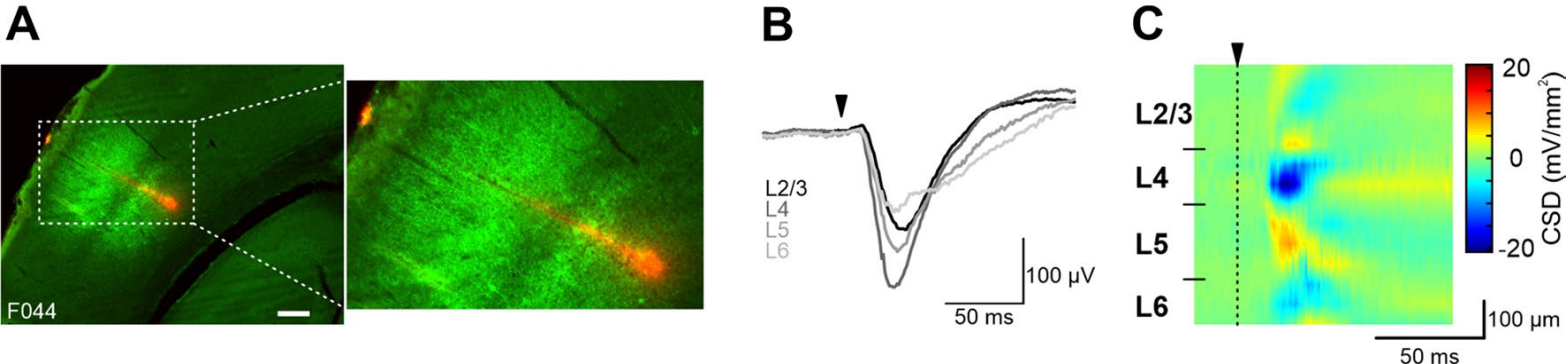
Illustration of the experimental approach and example recording traces. (**A**) Left: Coronal slice of mouse barrel cortex showing opsin expression (green) and probe penetration (DiI, red). Scale bar: 200 µm. Right: Magnification of the enclosed area in the left picture. (**B**) Example LFP traces from one mouse (low-pass filtered at 250 Hz). Shown are selected channels from each cortical layer, averaged over 24 W1 deflections. The triangle indicates stimulus onset. (**C**) Example CSD profile showing allocation of cortical layers to electrode channels (32 channels, 25 µm inter-electrode spacing).

Opsin expression around the area of probe insertion was confirmed histologically (Fig. 1A). The extent and specificity of opsin expression across cortical layers was assessed by counting the number of cells expressing ArchT-GFP and/or PV. Overall, opsin expression was highest in layers 2/3 and lowest in layer 4 (Supplemental Fig. 1). The minimum specificity of ArchT expression (measured by the overlap of ArchT-GFP and PV fluorescence) reached 65% in layers 2/3, 51% in layer 4 and 39% in layers 5/6, with marked heterogeneity between individual slices (Supplemental Fig. 1J).

### Local field potentials

In all animals, whisker deflection led to a clear LFP response that was visible in all cortical layers (Fig. 1B). We used the LFP response to compute current source density (CSD) profiles from which we could infer the position of each electrode with respect to its cortical layer position (Fig. 1C, see Methods for details).

**Figure 2:**
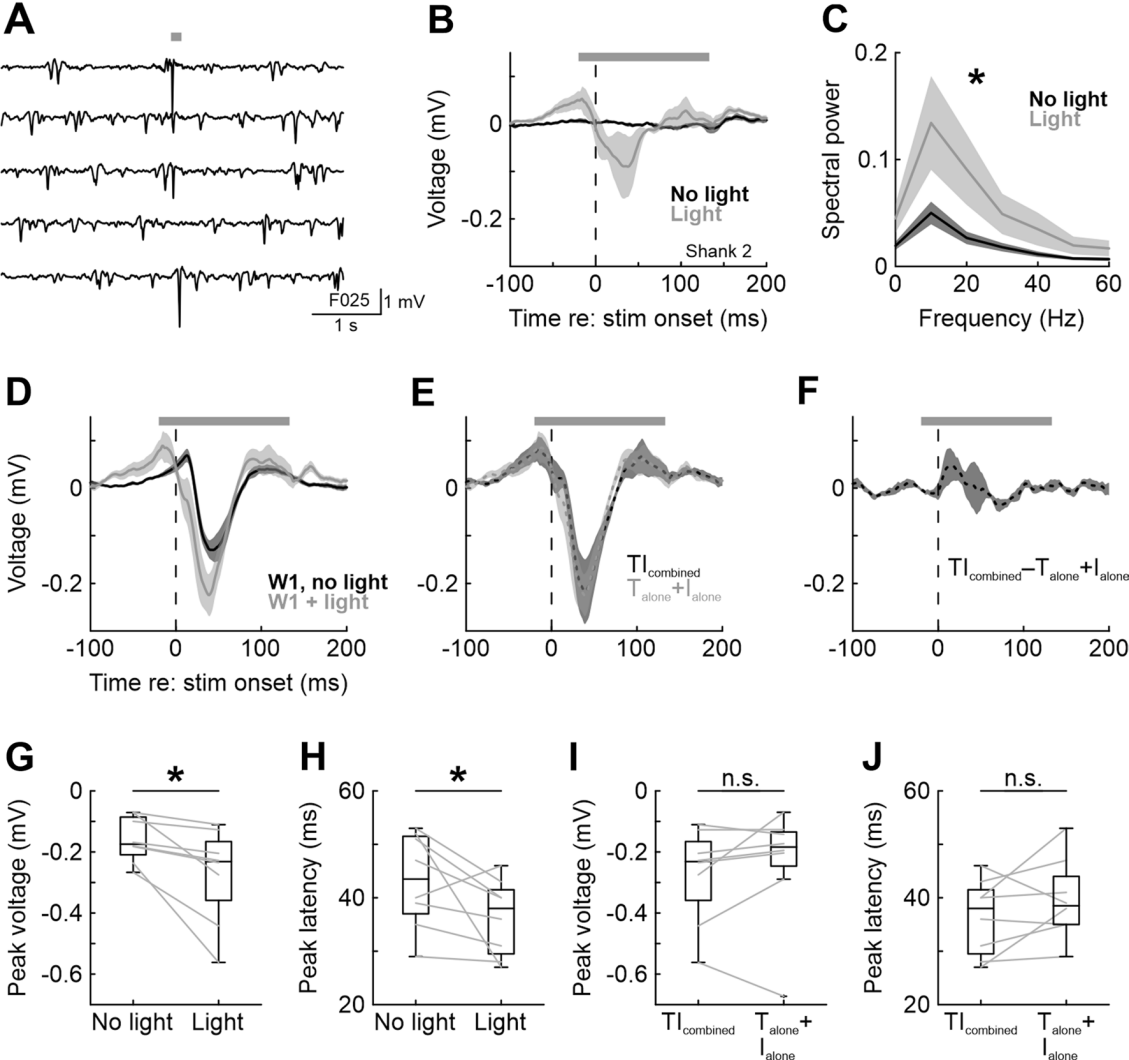
Optogenetic inhibition of PV+ interneurons affects ongoing brain activity and increases LFP responses to single-whisker deflections. (**A**) Five example traces from one animal showing background activity and light-evoked negative deflections of the LFP. The duration of light application is indicated by the horizontal gray bar. (**B**) Average LFP activity with and without light application (each trace averaged over n = 8 mice, 25 trials per stimulus, shading represents SEM over mice). In this and all other LFP panels of Fig. 2, LFP traces were high-pass filtered at 4 Hz to remove light artifacts (see Methods). (**C**) Spectrograms of 100 ms LFP segments during light application (light on for 153 ms, first 20 and last 33 ms omitted from analysis to exclude laser onset and offset artifacts). Light increases LFP power across all frequencies. (**D**) Average LFP responses in deep L2/3 to a single 30 Hz cosine pulse to whisker W1 with and without light application. (**E**) Comparison of the combined effect of whisker deflection and light application (dark gray, same trace as in (**D**) and the calculated summation of the LFP responses to whisker deflection and light application in isolation (light gray). (**F**) Difference between the two traces in E. (**G**) Boxplot of average peak amplitudes within the first 60 ms after stimulus onset; data from individual mice shown as gray lines. Corresponds to the traces shown in panel D. Asterisks in this and other panels indicate significant (*p* < 0.05) differences; n.s., not significant. In this and all other figures of the main text, boxplots were constructed such that the box ranges from the 1st to the 3rd quartile of the data distribution, the horizontal line represents the median, and the whiskers encompass either the entire data distribution or extend until 1.5 times the interquartile range, whichever is shorter. In the latter case, more extreme data points (outliers) are shown as + signs. (**H**) Same as in G, but for peak latency. (**I**) As in G, but calculated from the data shown in E. (**J**) As in I, but for peak latency.

First, we investigated the effect of optogenetic silencing on neural activity without concomitant whisker stimulation. Background activity under medetomidine/midazolam/fentanyl (MMF) anesthesia consisted of spontaneously occurring negative deflections in the LFP, which also could be elicited by light application in a fraction of trials (Fig. 2A,B).

As we have shown previously^26^, optogenetic silencing of PV+ interneurons led to an increase in LFP power across frequencies collapsed from 0 to 60 Hz (Fig. 2C; sign-rank test, *p* = 0.023, n = 8 electrodes in deep L2/3, one electrode from each mouse; see Methods for details). Similarly, the LFP response to whisker deflection was larger upon optogenetic inhibition of PV+ interneurons. The peak of the evoked response increased on average by 78% under illumination relative to the control condition (sign-rank, *p* < 0.01, n = 8; Fig. 2D,G), with a small but significant change in the latency of the response peak (40 vs. 46 ms with and without light, respectively; sign-rank, *p* < 0.01, n = 8, Fig. 2H).

Since light application alone resulted in a LFP response profile resembling that evoked by an isolated whisker deflection (Fig. 2B), we asked whether the effect of PV+ inhibition on the stimulus-evoked response could be explained as the linear sum of the two isolated effects. We therefore compared the LFP response recorded during tactile stimulation under illumination (TI_combined_; gray trace in Fig. 2D) with the summed LFP responses recorded during illumination alone (I_alone_; gray trace in Fig. 2B) and the tactile stimulation alone (T_alone_; i.e., without concomitant light application; dark gray trace in Fig. 2D). The result is shown in Fig. 2E and displays a striking similarity of the two traces. Consequently, the time-resolved difference of the two traces was close to 0 mV throughout the stimulation epoch (Fig. 2F). Moreover, there was no significant difference between peak responses and peak latencies between the two traces (Fig. 2I,J; *p* = 0.172 and *p* = 0.313, respectively; sign-rank tests, n = 8 in both cases). Thus, whisker deflection and light-mediated inhibition of PV+ interneurons exert additive and independent (rather than interacting) effects on ongoing neuronal activity as measured via LFPs.

**Figure 3:**
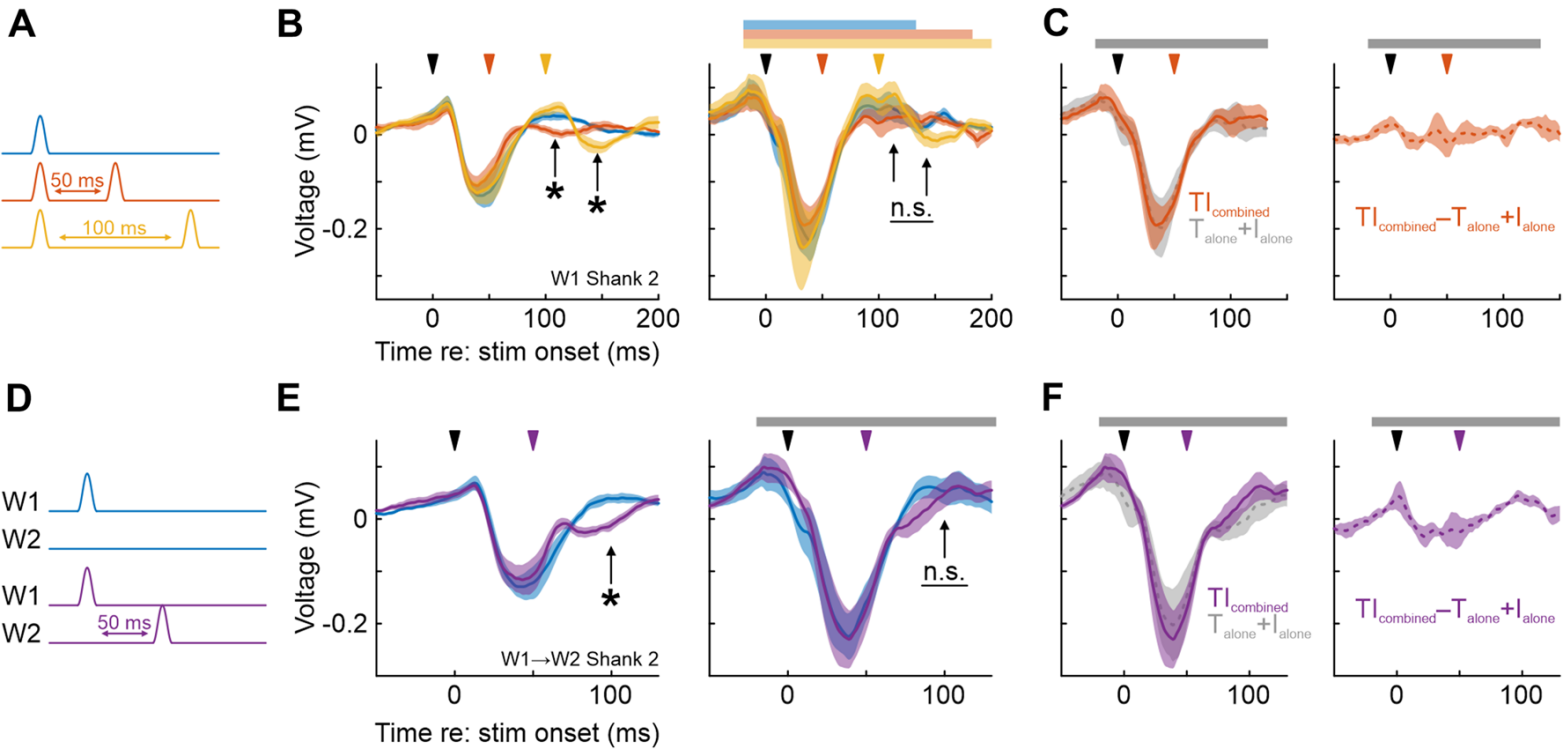
Optogenetic inhibition of PV+ interneurons increases LFP responses to multiple whisker deflections. (**A**–**C**) LFP responses to subsequent deflections of the same whisker after 50 ms or 100 ms. (**A**) Sketch of the stimulation protocol. (**B**) Left: Average LFP responses to the three stimuli illustrated in the sketch, without light application. Arrows indicate LFP components representing the 2nd stimulus pulse. Right: As in the left panel, but during light application. Horizontal bars indicate period of light application. (**C**) Left: For 50 ms interpulse-interval, summation of the two LFP responses from whisker deflection alone and light application alone (gray) and when recorded under both tactile and light stimulation (red, same as in panel B). Right: Differences between the traces shown in the left panel. (**D**–**F**) LFP responses to subsequent deflections of adjacent whiskers (W2 50 ms after W1). D) Sketch of the stimulation protocol. (**E**) Left: Average LFP responses to the two stimuli without light. Right: As in the left panel, but with light application. (**F**) Left: For 50 ms interpulse-interval, summation of the two LFP responses from whisker deflection alone and light application alone (gray) and when recorded under both tactile and light stimulation (lilac, same as in panel E). Right: Differences between the traces shown in the left panel.

During free exploration, adult mice exhibit rhythmic movements of their vibrissae (“whisking”;^27^. This movement results in the transformation of the shape of objects (a purely spatial pattern) into complex spatiotemporal patterns of whisker stimulation. In a simplified scheme, a single whisker sweeping past two vertical poles separated by a small distance will transform the spatial distance parameter into a temporal interval, which then elicits a neural representation of the distance between the poles. Are PV+ interneurons important for the representation of these temporal intervals? To answer this question, we applied two consecutive whisker deflections at two different inter-deflection intervals of 50 and 100 ms and compared the observed LFP responses to that resulting from the single deflection of the same whisker (Fig. 3A).

Without optogenetic modulation, the LFP response profiles of the three stimuli were clearly different (Fig. 3B, left panel, arrows): depending on the inter-stimulus interval, the second whisker deflection significantly affected the LFP response after about 30–50 ms following deflection onset (arrow heads, sign-rank of minimum voltage values, single pulse vs. two pulses separated by 50 ms, analysis window from 90 to 130 ms: *p* = 0.003, n = 8 for single vs. two pulses separated by 100 ms, analysis window from 130 to 170 ms: *p* = 0.016, n = 8). Interestingly, these differences vanished when PV+ interneurons were inhibited (Fig. 3B, right panel, difference between LFP traces not significant, sign-rank tests, both *p* > 0.10; n = 8).

Again, we asked to what extent the effect of light-mediated PV+ inhibition is additive and independent of the effect of whisker stimulation. To address this question, we performed the same analyses as shown in Fig. 2, comparing LFP responses to concomitant tactile and light stimulation to the linear summation of the LFP responses recorded under tactile and light stimulation alone. In good agreement with our previous results, this analysis again pointed to an additive effect of the two manipulations, as the recorded and constructed LFP traces were highly similar and did not exhibit significantly different response peaks (Fig. 3C, sign-rank, *p* = 0.11, n = 8).

During whisker sweeps, adjacent whiskers contact objects in temporal succession. To investigate the representation of such spatiotemporal patterns in barrel cortex, and to examine their reliance on PV+ interneurons, we stimulated neighboring whiskers in close temporal proximity (inter-deflection interval of 50 ms, similar as occurs during object palpation; Fig. 3D). We found that 50-ms intervals led to a clearly discernible difference of the LFP response compared to a single whisker deflection (Fig. 3E, analysis as in 3B, *p* < 0.01). Optogenetic silencing affected the response profile, again abolishing the significant difference (sign-rank, *p* = 0.55, n = 8). Addressing the independence of the effects of PV+ inhibition and whisker deflection, we repeated the analysis already shown in Fig. 2E,F, and we again obtained similar results—observed and predicted traces were highly similar, although now there was a small but significant difference regarding peak amplitudes (sign-rank, *p* = 0.008, n = 8; Fig. 3F).

In sum, optogenetic inhibition of PV+ interneurons (1) increased LFP power across all frequency bands, (2) increased the LFP peak response to whisker deflection, (3) slightly decreased LFP peak latency, (4) impaired the representation of temporal sequences of whisker deflections such that a second whisker deflection after 50 ms could not be detected anymore, and (5) additionally affected the representation of the deflection of an adjacent whisker after 50 ms. (6) Most importantly, PV+ inhibition seems not to affect sensory processing per se, as the LFP response observed during both tactile stimulation and PV+ inhibition closely matched the sum of the two manipulations observed in isolation, demonstrating an independent additive effect of these two processes.

In the next section, we will present the results of single-neuron recordings under identical stimulation conditions.

### Single neurons

Overall, we recorded 98 single neurons from all neuronal somata-containing cortical layers (2 neurons from layer (L) 2/3, 19 neurons from L4, 39 neurons from L5, 38 neurons from L6). 20 of these 98 neurons were classified as putative fast-spiking inhibitory interneurons (INH) on the basis of spike width (20%; see Methods), the remainder as putative excitatory neurons (EXC).

**Figure 4:**
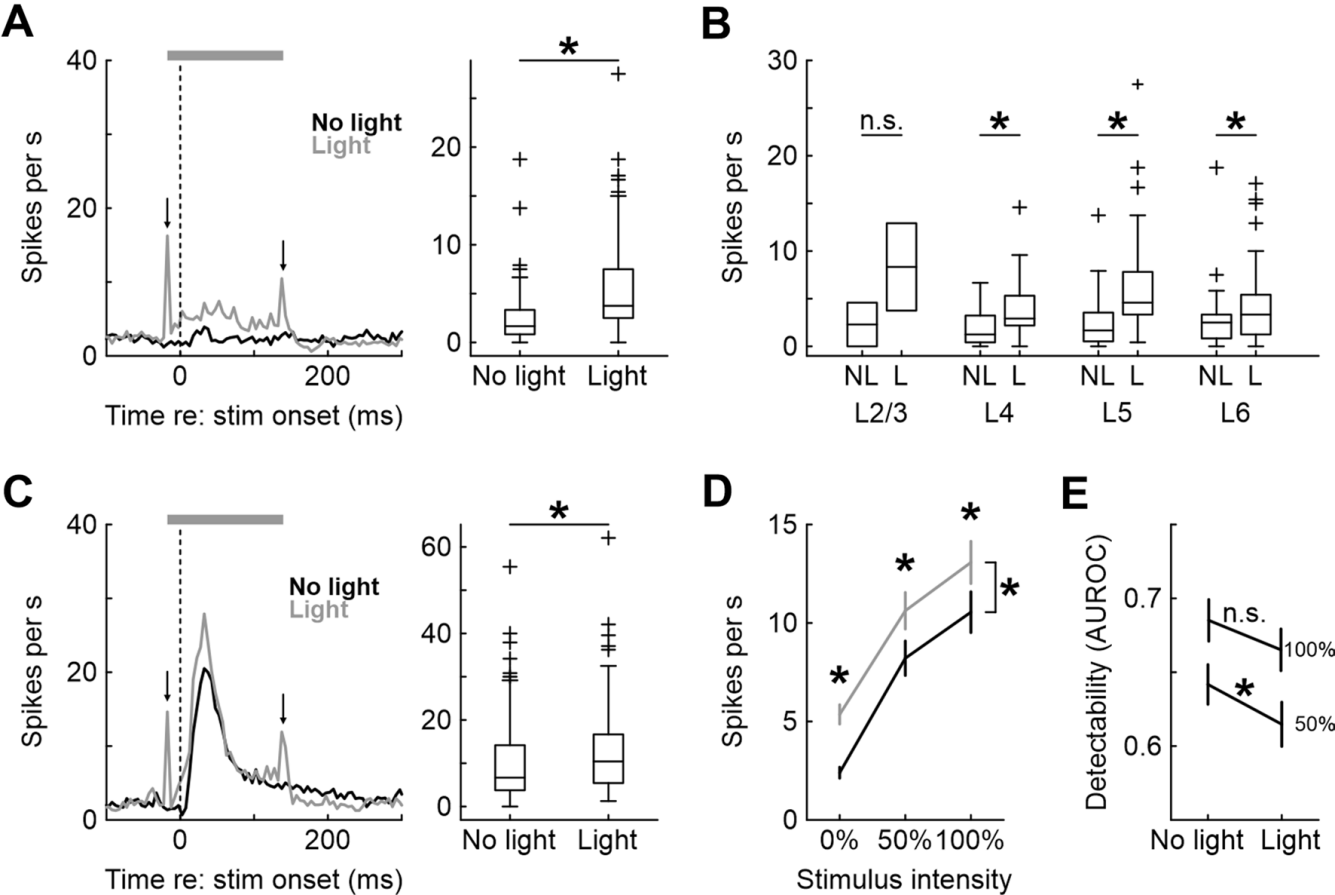
Effect of optogenetic inhibition of PV+ interneurons on spontaneous and single-whisker stimulus evoked activity in single neurons. (**A**) Left: Mean PSTH across n = 98 single units with and without light application (gray and black lines, respectively). In this and the following panels, downward pointing arrows indicate light onset and offset artifacts, gray horizontal bar denotes the period of light application, and dashed vertical line depicts stimulus onset. Bin size is 5 ms. Right: Boxplots for mean spontaneous firing rates of n = 98 single units across all cortical layers comparing activity with and without light application. (**B**) Spontaneous activity with (L) and without light application (NL) for all single units shown separately for cortical layers. (**C**) Left: Mean PSTH across all single units in response to a maximum-amplitude W1 deflection, with and without light application. Right: Boxplot comparing mean neuronal response to the stimulus. (**D**) Left: Mean neuronal response to three different stimulus intensities, with and without light. Error bars represent SEM. (**E**) Detectability (AUROC) across all single neurons for 50% and 100% stimulus amplitudes compared to the null stimulus, both with and without light application.

Light application increased spontaneous firing rate by 125% (from 2.4 ± 0.3 [mean ± SEM here and elsewhere] to 5.4 ± 0.3 Hz, sign-rank, *p* < 10^–11^; n = 98 single units; Fig. 4A). This was mostly driven by putative excitatory neurons: 25 of 78 EXC neurons significantly increased firing during light stimulation, but only 2 of 20 INH neurons did so (rank-sum test for each neuron comparing all 24 light on to all 24 light off trials, *p* < 0.05). None of the recorded neurons significantly decreased firing rate. This is in agreement with our previous report^26^ with a larger data set, in which only 6/72 INH neurons decreased firing, while 10/72 INH neurons increased firing.

A significant net increase of firing rate during light application was evident in all cortical layers (L2/3: 2.3 ± 2.3 vs. 8.3 ± 4.6 Hz, L4: 1.9 ± 0.4 vs. 4.1 ± 0.8 Hz, L5: 2.4 ± 0.4 vs. 6.5 ± 0.9 Hz, L6: 2.7 ± 0.5 vs. 4.6 ± 0.7 Hz; sign-rank tests, L2/3: *p* = 1, n = 2, L4: *p* = 0.001, n = 19; L5: *p* < 10^–6^, n = 39; L6: *p* < 10^–3^, n = 38; Fig. 4B). As expected, neurons fired more during W1 stimulation when PV+ interneurons were inhibited (10.5 ± 1.0 vs. 13.1 ± 1.1 Hz in the first 100 ms after stimulus onset, sign-rank *p* < 10^–5^, n = 98; Fig. 4C). This was true for both EXC and INH neurons (EXC: 10.2 ± 1.0 vs. 13.0 ± 1.0 Hz, n = 78; INH: 12.0 ± 3.4 vs. 13.4 ± 3.5 Hz, n = 20, sign-rank, both *p* < 0.05). Dissecting this effect by layers, we found significant increases only in infragranular layers (sign-rank, L5: 10.6 ± 1.9 vs. 13.6 ± 2.0 Hz, *p* < 10^–3^, n = 39; L6: 9.2 ± 1.3 vs. 11.9 ± 1.3 Hz, *p* < 10^–5^, n = 38), but not in L4 (12.7 ± 2.2 vs. 12.7 ± 2.3 Hz, *p* = 0.84, n = 19; sign-rank tests). In subsequent analyses, we will mostly present results analyzed across cell types and cortical layers because we found that cell samples of 20 or less rarely proffered sufficient statistical power to yield significant results.

Does PV+ neuron inhibition affect neuronal activity in an additive or multiplicative manner? If PV+ neurons controlled the gain of the neuronal response, we would expect that the number of evoked spikes is increased during light application by a constant factor (i.e., multiplied). Alternatively, the effect of PV+ neurons might be additive, implying a constant offset. Figure 4D plots mean firing rate for baseline activity compared to 50 and 100% W1 stimulation amplitude for light and no-light conditions. Visual inspection suggests that PV+ neuron inhibition increases neural activity by a constant offset, and this was confirmed statistically for all neurons: light application significantly increased firing rate at each stimulus intensity (sign-rank, all n = 98 and *p* < 10^–5^) by a similar amount (3.0 ± 0.4, 2.4 ± 0.5, and 2.5 ± 0.6 Hz for 0, 50, and 100%, respectively; Friedman test on n = 98 difference values, *p* = 0.91). This was also true when EXC and INH were analyzed separately (Friedman test, *p* = 0.96 (n = 78) and *p* = 0.32 (n = 20), respectively) and for each individual layer (Friedman test, all *p* > 0.2, n was 2, 19, 39, and 38 for layers 2/3, 4, 5, and 6, respectively).

This observation is important: An unspecific additive increase in firing rate which manifests regardless of stimulation condition directly affects the signal-to-noise ratio (SNR) of the neuronal signal. Adding a constant offset to both the numerator and the denominator decreases the ratio (e.g., for SNR of 2 = 2/1, adding 2 yields SNR = 4/3 = 1.33). The fact that PV+ neurons inactivation does just that suggests that PV+ neuron activity may be important to generate a faithful (i.e., high-SNR) representation of tactile input. In line with this hypothesis, we found that stimulus detectability (AUROC) for 50% intensity was significantly reduced by light application (stimulus vs. baseline, AUROCs 0.64 ± 0.01 and 0.61 ± 0.01 under no-light and light conditions, respectively, sign-rank *p* = 0.028, n = 98; Fig. 4E). The difference in discriminability for the 100% stimulus intensity was of similar magnitude but failed to achieve statistical significance (AUROCs 0.69 ± 0.01 and 0.67 ± 0.01, respectively; sign-rank, *p* = 0.097, n = 98). The same results pattern was found when restricting the analysis to the 78 EXC neurons separately (50% intensity, no light: 0.64 ± 0.01, light: 0.62 ± 0.02; sign-rank, *p* = 0.036; 100% intensity, no light: 0.69 ± 0.02, light: 0.67 ± 0.02, sign-rank, *p* = 0.18), but not when analyzing only the 20 INH neurons (50% intensity, no light: 0.63 ± 0.03, light: 0.60 ± 0.03; sign-rank, *p* = 0.53; 100% intensity, no light: 0.68 ± 0.04, light: 0.65 ± 0.03, sign-rank, *p* = 0.28). Of note, INH neurons showed similar reductions of detectability as EXC neurons in numerical terms, and it is therefore most likely that the small sample size is insufficient to detect a significant effect. It is therefore not possible to say that the reduction in detectability is restricted to EXC neurons.

**Figure 5:**
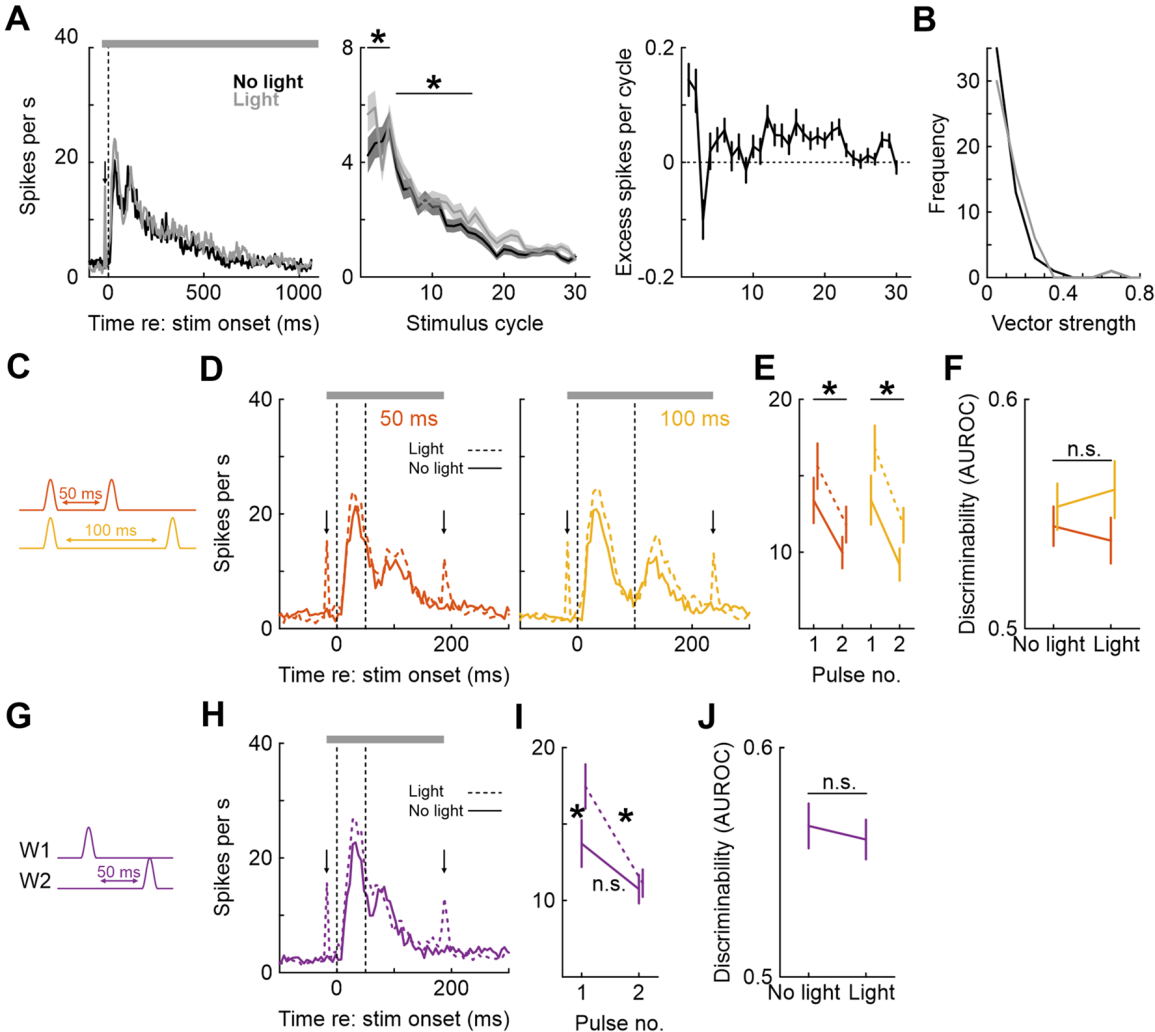
Effect of optogenetic inhibition of PV+ interneurons on vibrotactile and multiple whisker stimulation in single neurons. (**A**) Left: Mean PSTH across all units in response to a 1 s 30 Hz cosinusoidal vibrotactile stimulus, with and without light. Middle: Mean firing rate per stimulus cycle, shading represents SEM. Right: Number of additional (excess) spikes fired during light application (i.e., the difference between firing rates under light application and without light). Error bars represent SEM. (**B**) Histograms of vector strengths for all units. (**C**–**F**) Effect of PV inhibition during single-whisker double-pulse stimulation on neuronal activity. (**C**) Illustration of the stimulus protocol. (**D**) Left: Mean PSTHs in response to stimulation, with (dashed lines) and without (solid lines) light application for 50 ms interpulse intervals. Vertical dashed lines denote deflection onsets. Right: Same, but for 100 ms interpulse intervals. (**E**) Mean firing rates (and SEM) during the first and second stimulus pulses, for 50 and 100 ms interpulse intervals, with and without light application. (**F**) Neurometric discriminability (AUROC) of 50 and 100 ms double pulse stimulus compared to single W1 deflections. (**G**–**J**) Effect of PV inhibition during two-whisker consecutive stimulation. (**G**) Illustration of stimulus protocol. (**H**) Mean PSTH in response to W1- > W2 stimulation (50 ms interpulse interval), with and without light. Conventions as in D. (**I**) Mean firing rate in response to each of the two deflections, with and without light. (**J**) Neurometric discriminability (AUROC) for the double-pulse stimulus compared to a single W1 deflection, averaged over all units.

To mimic the situation when a sweeping whisker is in constant contact with a surface, we applied a 1-s long vibrotactile stimulus (30 Hz sinusoidal vibration) to W1. This stimulus reliably activated neurons throughout the stimulation epoch (Fig. 5A). Light application increased firing rate not only during the phasic onset response (sign-rank, first 100 ms: 12.8 ± 1.3 vs. 14.8 ± 1.3 Hz, *p* < 0.001, n = 98), but also during the steady state (150–500 ms, 7.2 ± 0.8 vs. 8.3 ± 0.8 Hz, *p* < 10^–4^, n = 98), consistent with the LFP data (Fig. 2C) showing enhanced spectral power. In general, the effect of light led to a step-like increase in firing rate which however was slightly more pronounced during the early response epoch (increase ~ 2 Hz early vs. ~ 1 Hz late, sign-rank *p* = 0.017, n = 98; Fig. 5A, middle and right panels).

Upon sinusoidal whisker deflection, barrel neurons have been shown to phase-lock to stimulus cycles^28^, which is believed to subserve the representation of whisker vibration^29^. We next asked whether the degree of phase locking was affected by optogenetic silencing of PV+ interneurons. Average vector strength^30^ of all units which fired at least 50 spikes during the steady-state analysis period (24 trials, 11 sine wave cycles from 133.3 to 500 ms post stimulus onset, 53 neurons) was similar during light on and light off conditions (0.107 ± 0.015 vs. 0.109 ± 0.015, sign-rank *p* = 0.74, n = 98), suggesting that PV+ neuron inhibition does not affect or impair neuronal phase-locking to stimulus cycles (Fig. 5B).

To investigate the representation of stimulus sequences, we stimulated the same whisker twice with different inter-pulse intervals of 50 and 100 ms (Fig. 5C). Stimulation resulted in a biphasic response profile (Fig. 5D) for both intervals. The first pulse consistently resulted in a larger neuronal response than the second pulse (no light: 13.4 ± 1.5 vs. 10.0 ± 1.0 Hz in 0–60 vs. 60–120 ms after stimulus onset for the 50 ms double pulse, 13.4 ± 1.6 vs. 9.2 ± 1.1 Hz for the 100 ms double pulse; light: 15.6 ± 1.5 vs. 11.8 ± 1.2 Hz and 16.8 ± 1.5 vs. 11.8 ± 1.1 Hz for the 50 and 100 ms double pulse stimuli), and this was true for both light off and light on conditions (sign-rank, all *p* < 0.01, all n = 98) (Fig. 5E). Again, light application increased neuronal activity non-specifically, i.e. for both pulses for each stimulus (sign-rank, all *p* < 0.003, all n = 98).

We next asked how well evoked spike counts could predict stimulus identity. We found that discriminability between the 50 ms double pulses and a single pulse was similar to that between 100 ms double pulses and a single pulse (AUROCs 0.54 ± 0.01 and 0.55 ± 0.01, sign-rank, *p* = 0.26, n = 98), although both were significantly different from 0.5 (sign-rank, both *p* > 0.05, n = 98 in both cases; Fig. 5F). Mean discriminability (AUROC) was unaffected by light application (50 ms: AUROC = 0.54 ± 0.01, 100 ms: AUROC = 0.56 ± 0.01, sign-rank, both *p* values > 0.05, both n = 98).

Extending this to the subsequent deflection of adjacent whiskers (W1 always first; Fig. 5G) gave essentially the same result (Fig. 5H,I): first pulses evoked higher firing rates than second pulses (analysis as for Fig. 5E; no light: 13.7 ± 1.5 vs. 10.7 ± 0.9 Hz in 0–60 vs. 60–120 ms after stimulus onset, light: 17.4 ± 1.5 vs. 11.1 ± 0.9 Hz), although this was statistically significant only for the light on condition (sign-rank, *p* = 0.24 and *p* < 0.01 for light off and light on, respectively; n = 98 in both cases). Light significantly increased neuronal activity for the first (W1) deflection (sign-rank, *p* < 0.01, n = 98) but not for the subsequent W2 deflection (sign-rank, *p* = 0.07, n = 98). Again, mean discriminability was unaffected by light application (no light: AUROC 0.57 ± 0.01, light: AUROC 0.56 ± 0.01; sign-rank, *p* = 0.93, n = 98; Fig. 5J).

To summarize, optogenetic inhibition of PV+ interneurons (1) increased firing rate regardless of stimulus strength, indicative of an additive rather than multiplicative effect of PV+ neurons, (2) affected neuronal discriminability for a moderate-intensity stimulus, (3) was most pronounced during the first 100 ms of a 1-s vibrotactile stimulus, (4) did not change phase-locking of spikes to stimulus cycles, and (5) did not affect neurometric discriminability of single vs. double-pulse whisker stimuli.

## Discussion

Previous in vitro and in vivo studies already demonstrated in various cortical areas the pivotal role of PV+ GABAergic interneurons in modulating spontaneous and sensory-evoked activity (for review^9,16^ and in the control of behavior (for review^31^). Furthermore, it has been suggested that PV+ interneurons are involved in the early manifestation of various neurological and psychiatric disorders (for review^32^). However, the role of PV+ interneurons in controlling the spatiotemporal representation of a defined sensory stimulus in the adult cerebral cortex is not fully understood. Using a combination of two-photon calcium imaging and optogenetic inhibition of PV+ interneurons in mouse visual cortex in vivo, Agetsuma and colleagues demonstrated that PV+ cells regulate network dynamics both temporally and spatially, thereby making neocortical ensembles more distinct from each other^33^. Here, we used multi-electrode arrays to record with high temporal resolution LFPs and single units in all layers of the barrel cortex of anesthetized adult mice. ArchT-expressing PV+ interneurons were optogenetically silenced using experimental protocols established in our previous study^26^.

In our present study, the specificity of opsin expression in PV interneurons particularly in infragranular layers has been rather low, despite employing the cre-LoxP system, which should restrict expression to neurons or cells expressing cre-recombinase^19^. We would like to stress, that an analysis of both the numbers of opsin-expressing cells as well as the specificity of expression is critical for any study involving the effects of optogenetics on functional circuits (for review^22^). Our current histological analysis most likely provides only a lower bound on specificity, which may potentially considerably underestimate its value. First, specificity as measured here can only provide a lower bound estimate on the real value, because not all PV+ interneurons will actually stain positive for PV. Moreover, fluorescence intensity may have diminished between histological staining and cell quantification. Second, in our previous publication not a single neuron (out of 441) co-stained for PV and CaMKII^26^, which strongly argues against any expression in excitatory neurons. This fits with our observations that not a single excitatory neuron significantly decreased firing rate under illumination, as would be expected if ArchT were expressed in these neurons to a substantial extent. Third, we replicated several key observations of our previous paper, among them an unspecific increase of power in all LFP frequency bands, evocation of negative LFP deflections during light application, an increase in the evoked LFP peak response during light application, as well as an overall increase in firing rate across all layers and subclasses of neurons. In sum, we are confident that the physiological effects described in this paper arise mainly from light-mediated inhibition of PV+ interneurons.

In good agreement with our previous report^26^, optogenetic inhibition induced a pronounced increase in the LFP power during spontaneous activity, across all frequencies from theta to gamma rhythm. This increase in spontaneous firing was almost exclusively mediated by putative excitatory neurons and could be observed in all cortical layers, demonstrating the powerful brake of soma-targeting PV+ cell-mediated inhibition on spontaneous activity. Our observations are further in good agreement with previous in vitro and in vivo observations in barrel cortex of adult mice. During Up states, PV+ interneurons are by far the most active cell type^34^, and during Down states PV+ cells receive suprathreshold activation via spontaneous thalamic activity thereby causing inhibition of barrel cortical networks^35^. Thus, PV+ interneurons play a central role in the regulation of cortical spontaneous activity.

Optogenetic silencing of PV+ interneurons did not only cause a rise in spontaneous network and pyramidal cell activity, but also a significant increase in LFP and single unit responses to single whisker stimulation. We assessed the role of PV+ interneurons in the spatiotemporal representation of sensory stimuli by stimulating one or two whiskers at different, physiologically relevant intervals and simultaneously silenced PV+ cells. Double-stimulation of the same whisker at 50 or 100 ms interval elicited a small negative LFP response after about 30–50 ms. This LFP component was partially blocked when PV+ interneurons were inhibited, indicating that these late responses are influenced by GABAergic inhibitory mechanisms and that PV+ cells sharpen the temporal discrimination of a sensory stimulus. A similar observation could be obtained when two adjacent whiskers were stimulated at an interval of 50 ms, resembling the spatiotemporal activation pattern during active whisking. During optogenetic inhibition of PV+ interneurons, the stimulation of the adjacent whisker could no longer be detected in the LFP recording, demonstrating that PV+ cells have a major impact on the spatial resolution of the sensory stimulus. In upper layers, these effects are most likely mediated by L2/3 PV+ interneurons, which are activated from excitatory L4 spiny neurons following whisker stimulation (for review^7^). However, the increase in overall firing rate induced by optogenetic inhibition was visible across all layers (Fig. 4B), suggesting that PV+ interneurons in all layers shape the spatial and temporal resolution of the sensory stimulus in the primary somatosensory cortex.

When single units were further analyzed in their response pattern to single whisker stimulation, we observed in all cortical layers, but with the exception of L4, an increase in the mean firing rate when PV+ interneurons were silenced. These data suggest that PV+ cell-mediated inhibition prevents pyramidal neurons from firing. It has been previously demonstrated that whisker stimulation elicits mostly subthreshold responses and only ~ 10% of upper layer pyramidal neurons spike during active touch^36,37^. Our data indicate that removal of this PV+ cell-mediated inhibitory brake unleashes pyramidal cells and causes an increase in their discharge rate to sensory stimulation. During optogenetic inhibition of PV+ interneurons, single units in all cortical layers also revealed a constant and linear increase in their mean firing rate to single whisker stimulation at different stimulus intensities. These data are in good agreement with previous observations in mouse visual cortex, where optogenetic inhibition of PV+ interneurons induced a significant increase in the spike rate of L2/3 pyramidal neurons to visual stimuli of different contrast^38^. Of note, we could observe these strong effects despite putatively only silencing a minor fraction of all PV+ interneurons^26,39^. We also found that detectability of a sensory stimulus with moderate (50%) intensity was significantly improved by the action of PV+ cells. All these results demonstrate that PV+ -mediated GABAergic inhibition of cortical pyramidal neurons during sensory processing modulates cortical activity and improves signal-to-noise ratio of the sensory input^40^.

Mice whisk at frequencies of 9–16 Hz during locomotion and > 20 Hz during periods of intense whisking^41,42^. Since this natural whisking behavior results in cortical activation over longer time periods than with single or double stimuli, we also assessed the responses of barrel cortical neurons to sinusoidal 30 Hz vibration of a single whisker. Previous recordings in barrel cortex of anesthetized rats demonstrated phase-locked 1:1 responses to vibratory frequencies of up to ~ 300 Hz^28^. Our recordings demonstrate that neurons largely follow the 30 Hz stimulation during the phasic onset response (first 100 ms) and subsequently decrease their firing rate. Optogenetic inhibition of PV+ interneurons had a small, but significant effect only on the early response and on EXC neurons only. The degree of phase-locking to stimulus cycles was not influenced, neither for EXC nor for INH neurons.

In conclusion, our multi-electrode recordings in barrel cortex of adult anesthetized mice suggest that the dampening effect of PV+ interneurons on ongoing neuronal activity is important for sharpening the spatio-temporal representation of physiologically relevant sensory stimuli.

## Methods

Animal experiments were approved by the local German ethics committee (Landesuntersuchungsamt Rheinland-Pfalz Koblenz, #23 177-07/G19-1-085). This study is in accordance with the ARRIVE guidelines. Male PVcre mice (B6; 129P2-Pvalbtm1(cre)Arbr/J; The Jackson Laboratory, Bar Harbor, Maine, USA), aged 4–6 weeks were chosen for experiments. Animals were kept in cages in groups of 2–3 and had ad libitum access to food and water. All procedures followed European and German laws (European Communities Council Directive, 86/ 609/ECC).

### Animal surgery for intrinsic optical imaging and virus injection

The localization of the whisker-related barrel column was achieved through optical imaging of intrinsic signals. Mice were sedated with 5% v/v isoflurane in ambient air in a closed chamber and injected i.p. with a mixture of Medetomidin (0.5 mg/kg), Midazolam (5 mg/kg), and Fentanyl (0.05 mg/kg). The depth of anesthesia was evaluated based on the breathing pattern and the toe pinch reflex. Eyes were covered by ophthalmic ointment to prevent drying. Carprofen (5 mg/kg) was administered to reduce the pain (s.c. injection 30 min after induction of anesthesia). A heating pad was laid beneath the mouse to maintain body temperature. After anesthesia induction, 0.2 ml of glucose (2.5% in saline) was injected to avoid dehydration.

After shaving the head, the skin was cleaned with 70% isopropyl alcohol pads for disinfection. Xylocaine gel (2%) was applied to the skin for local analgesia. An incision was performed on the midline and the skull was cleaned with saline. A custom-built metal head frame was glued to the skull to stabilize the animal’s head during the intrinsic optical imaging and subsequent virus injection. The frame was placed such that the barrel cortex (AP: −2 mm, ML: 3.5 mm relative to bregma) of the right hemisphere was accessible.

### Intrinsic optical imaging

The cortical surface was visualized through the intact bone by surface application of 1.5% agarose solution and addition of a glass coverslip on the top. The cortical blood vessel pattern was visualized with white light and used as reference image. At least three whiskers (C1, C2, B1) were stimulated using a miniature solenoid actuator positioned close to the whisker base. A whisker deflection lasted 10 ms and was repeated 20 times with an inter-stimulus interval of 25 ms. Trials of whisker stimulation bouts were repeated 30 times with an inter-trial interval of 20 s. Other whiskers were trimmed to avoid inadvertent stimulation of neighboring whiskers. Functional intrinsic signal images were computed as fractional reflectance changes relative to the pre-stimulus average. The pre-stimulus average was created identical to the stimulus sets but with the stimulator placed far off the whisker pad. The aimed barrel columns were identified by excitation light from red LED (630 nm; MRLED 625 nm, Thorlabs GmbH, Dachau, Germany) on the cortical surface while stimulating the whiskers. Images were collected with a MiCAM Ultima Master camera (Brain Vision Inc., Model MCULC3). The intrinsic signal images obtained for the barrel columns were then mapped to the blood vessel reference image and used to indicate the location for virus injection and also later on for the insertion of the multichannel silicon probe.

### Virus injection

Injections of viral solution were done under deep anesthesia immediately after intrinsic optical imaging. PV-Cre mice were placed on a warming pad and fixed with a custom-made metal head frame. Three small craniotomies were drilled tangentially around the center of the mapped barrel columns of interest (C1 and C2). Viral solution of rAAV2-FLEX-ArchT-GFP, containing 2 × 10^11^ viral particles per ml in PBS were delivered by a glass micropipette with microliter graduations (Hirschmann Laborgeräte, Eberstadt, Germany) pulled by a horizontal puller (Sutter Instruments, Novato, CA) and connected to a 50 ml syringe by manual pressure (for details see Fois et al. ^43^). Volumes of 750 nl of viral solution were slowly injected within 10 min first at 600 and thereafter at 300 μm cortical depth from the pia. After each injection, the pipette rested in place for another 10 min before slowly retracting. Electrophysiological recordings were performed no earlier than 4 weeks after virus injection. Viral transduction of PV+ interneurons with ArchT-GFP at the probe location was confirmed histologically in all animals (see Fig. 1A).

### In vivo multi-electrode recordings

On the day of the experiment, animals were anesthetized as described above. A cranial window of 1.5 × 1.5 mm was opened above the center of the mapped barrel columns with a dental drill (Ultimate XL-F, NSK, Trier, Germany) and covered with sterile injectable saline solution. Care was taken not to damage the dura. A silver wire was fixed over the cerebellum to serve as a ground electrode. Neural activity was recorded with a 2‐shank 64‐channel silicon probe (Cambridge NeuroTech, H-series Probe, 250 µm distance between shanks) inserted perpendicular into the barrel cortex targeting C1 or C2 columns identified with intrinsic optical imaging. Each of the two shanks (length 8 mm) contained 32 recording sites spaced 25 μm apart. Before insertion, the probe was labeled with DiI (1,1′‐dioctade‐cyl‐3,3,3′3′‐tetramethylindocarbocyanine; Molecular Probes, Eugene, OR) dissolved in 70% ethanol. The tracks of the shanks could be identified by DiI fluorescence in post-mortem histology. Data was continuously digitized at 20 kHz and stored offline on an extracellular recording system running MC_Rack software (Multi Channel Systems, Reutlingen, Germany).

### Optical stimulation setup

The light for excitation of ArchT was delivered by a 60 mW solid-state laser at 552 nm wavelength (Sapphire, Coherent, Dieburg, Germany). The laser was placed in a custom-built optical setup. The laser beam was coupled to a 400 μm multimode fiber with a numerical aperture of 0.39 (Thorlabs, Munich, Germany). To ensure reproducibility of the power density used, the output power at the end of the fiber was measured prior to each experiment with a power meter (Nova 2, Ophir, Newport, Irvine, CA). For excitation of ArchT, light intensity was adjusted to 111 mW/mm^2^ at the tip of the fiber. The light pulsing was controlled by a mechanical shutter (Uniblitz, Rochester USA) connected to a stimulator (Master8, A.M.P.I., Jerusalem, Israel). The fiber was positioned parallel to the electrodes and barely touched the cortical surface. Illumination always began 20 ms before stimulus onset and was maintained until 100 ms after stimulus offset. For example, for a single cosinusoidal 30 Hz deflection of 33 ms, total duration of illumination was 153 ms. The protracted onset of illumination was implemented to avoid contamination of sensory-evoked responses with light onset and offset artifacts.

### Whisker stimulation

Whisker stimulation was controlled by a power 1401 device controlled by Spike2 software (Cambridge Electronic Design, Cambridge, United Kingdom). Neighboring whiskers (C1 and C2) were inserted into open ends of two capillary tips which were glued to separate piezo actuators (Physik Instrumente, Karlsruhe, Germany). As shown in Table 1, different waveforms were transmitted to whiskers in random order during the experiment. Every stimulus was repeated 24 times; the inter-trial interval was 3 s. A whisker stimulation was realized by a sinusoidal waveform of 30 Hz (phase-shifted by 270° to ensure smooth stimulation onset; see Fig. 3B–E for example stimuli). The peak-to-peak amplitude of the stimulus was measured to be 560 µm for 100% intensity.

**Table 1:** Overview over stimuli (W1: (principal) whisker 1, W2: whisker 2).

1	Catch trial (no stimulation)
2	No whisker stimulation, only light (153 ms)
3	W1 stimulation, 50% intensity single pulse
4	W1 stimulation, 50% intensity single pulse + light
5	W1 stimulation, 100% intensity single pulse
6	W1 stimulation, 100% intensity single pulse + light
7	W1 stimulation, 100% intensity, two pulses w/ onsets separated by 50 ms
8	W1 stimulation, 100% intensity, two pulses w/ onsets separated by 50 ms + light
9	W1 stimulation, 100% intensity, two pulses w/ onsets separated by 100 ms
10	W1 stimulation, 100% intensity, two pulses w/ onsets separated by 100 ms + light
11	W2 stimulation, 100% intensity single pulse
12	W2 stimulation, 100% intensity single pulse + light
13	W1 stimulation, 100% 30 Hz vibration for 1 s
14	W1 stimulation, 100% 30 Hz vibration for 1 s + light
15	W1–W2 stimulation, both 100% w/onsets separated by 50 ms
16	W1–W2 stimulation, both 100% w/onsets separated by 50 ms + light

### Electrophysiological analysis

Local field potentials (LFPs) were obtained by digitally low-pass filtering the data at 250 Hz and downsampling to 1 kHz. For assignment of electrode channels to cortical layers, LFP responses to whisker deflection were subjected to current-source density (CSD) analysis as described previously (Fig. 1B,C;^26,44^). The presence of a short-latency current sink is a characteristic of layer 4 and served to distinguish this layer from L2/3 and upper L5. The boundary between L5 and L6 was determined on the basis of post-mortem histology and the known spatial extent of L5. For all further analyses, LFPs were additionally high-pass filtered at 4 Hz (Butterworth filter, 2nd order), because this filter setting was shown to eliminate the effects of the photoelectrical artifact on the LFP both in control recordings and in our previous study^26^ in which we systematically varied the cutoff-frequency of the filter. Highly similar results were obtained when the cutoff-frequency was set to 8 Hz. Note that we employed a noncausal high-pass filter (*filtfilt* in Matlab) which does not result in a phase shift and did not affect the estimation of peak amplitudes in our previous study, but changes the waveform of the evoked response such that it appears as if the response starts shortly before stimulus onset (compare Figs. 1B and 2D). Main analysis parameters (response peak, peak latency, average power) were extracted after averaging LFPs across trials for each stimulus.

For spike detection and sorting, data was imported into Plexon Offline Sorter (Plexon Inc., Dallas, Texas, USA). In the first step in order to reduce common noise, continuously recorded data from all electrodes was digitally referenced to the channel which was located most closely to the surface of the brain. Next, data was high-pass filtered at 250 Hz with a 4-pole Butterworth filter. Spike detection was performed using amplitude thresholding which was set − 6.5 times the standard deviation (SD) of the signal. The detected spikes were automatically sorted by the Valley Seeking algorithm and then manually curated to ensure the isolation quality of the sorted neurons. To be considered for further analysis, neurons had to fulfill several quality criteria: First, we required a signal-to-noise ratio (SNR) > 6 (SNR computed as the difference of the maximum and minimum peaks of the average waveform, divided by the 1% Winsorized standard deviation of the noise distribution (i.e., the 1% highest and lowest data points were removed and replaced by the most extreme remaining data points, which is a common procedure to obtain more robust estimates of the variance; see e.g.^45^). Second, less than 1.5% of interspike intervals were allowed to violate the refractory period of 2 ms. Putative excitatory and inhibitory units (EXC and INH, respectively) were identified on the basis of spike width, computed as full width at half minimum of the first peak of the spike waveform. This parameter showed a clearly bimodal distribution, and the cutoff was set at 150 µs. Main spike analysis parameters were firing rates computed over analysis windows of variable size (specified for each analysis). Laser onset and offset were accompanied by artifacts sometimes strongly resembling neuronal action potentials; inspection of channels without spikes confirmed that these artifacts were exclusively found at onset and offset. Therefore, analysis windows never overlapped with light onset and offset times (e.g., Fig. 4C, arrows). Vector strength was computed as described in^30^.

We used only nonparametric statistical procedures (Friedman test, Wilcoxon’s signed rank test, and the area under the receiver operating characteristic (AUROC) curve). For LFP data, all tests were based on one deep L2/3 electrode from one shank from each of eight mice. For spike data, all tests were either based on n = 98 single units from all layers, n = 78 EXC, or n = 20 INH neurons. All analyses were performed in Matlab (The Mathworks, Natick, USA) using custom-written code (see Matlab Central File Exchange #37,339 and #32,398 and^46^).

### Histology

After the experiment, the animal was deeply anesthetized with ketamine (120 mg/kg, Hameln Pharma, Hameln Germany) and xylazine (5 mg/kg, Rompun 2%, Bayer, Leverkusen, Germany) and perfused through the aorta with 0.2 M phosphate-buffered saline (PBS) and subsequently with 4% paraformaldehyde (PFA) for initial fixation. The brain was carefully removed from the skull and kept in 4% PFA for 24 h at 4 °C. Then the brain was washed three times with 0.1 M PBS and stored in 30% sucrose (in PBS) overnight. After rinsing with PBS, brains were sectioned in 30-μm thick coronal sections and prepared for immunohistochemistry. For the process of blocking and permeabilisation, after washing with 0.01 M PBS, slides were immersed in 7% normal donkey serum/0.8% Triton in 0.01 M PBS overnight at 4 °C. After another washing with PBS 0.01 M, slices were pre-treated with donkey anti-mouse IgG Fab fragment (1:20 in 0.01 M PBS, 715-007-003, Jackson ImmunoResearch via Dianova, Hamburg, Germany) for 2 h at room temperature.

Thereafter slices were rinsed with 0.01 M PBS and subsequently incubated with monoclonal mouse anti-parvalbumin IgG (1:1000, PV 235, Swant, Burgdorf, Switzerland), polyclonal rabbit anti-VGLUT2 (1:250, 135 402, Synaptic Systems, Göttingen, Germany) and polyclonal goat anti-GFP IgG (1:200, AB0020.200, Sicgen, Cantanhede, Portugal) in 2% bovine serum albumin with 0.05% azide and 0.3% Triton in 0.01 M PBS (3 days; 4 °C). Following a wash with 0.01 M PBS, slices were incubated with Cy3-conjugated polyclonal donkey anti-mouse IgG (1:200, 715-166-151, Dianova), Alexa Fluor 647-conjugated polyclonal donkey anti-rabbit IgG (1:200, 711-606-152, Dianova) and Cy2-conjugated polyclonal donkey anti-goat IgG (1:200, 705-225-147, Dianova) and DAPI (0.5 µg/ml, A4099, AppliChem, Darmstadt, Germany) in 2% bovine serum albumin with 0.05% azide (2 h; RT). The last step was a wash with 0.01 M PBS and embedding in Fluoromount-G (SBA-0100-01, SouthernBiotech via Biozol, Eching, Germany).

Imaging was conducted using an Apotome microscope (ZEISS, Jena, Germany). To determine the cell density of ArchT and the specificity of expression, four to five mosaic micrographs were taken from the injection site in coronal brain slices of five animals using a microscope camera (Axiocam 506 mono_ZEISS) and a 20 × objective (EC Plan-neofluar 20x/0.50 M27). After image acquisition, a maximum intensity projection of the measured z-stacks was created using the ZENlite (ZEISS, Jena, Germany) built-in function. All images were cropped for illustration purposes. Regions exhibiting homogeneous ArchT and PV expression were labeled using the freehand selection tool and the area was calculated in mm^2^ using the calibration tool of ImageJ^47^. The total volume was calculated by using the z-stack dimension and is reported in mm^3^. Further, we quantified ArchT and PV expression for each layer separately within the boundaries of the labeled area. Individual ArchT expression as well as ArchT/PV co-expression was counted and used to compute the specificity of co-expression.

## Supplementary Information

Supplementary Information.

## Data Availability

Data and code of this study are available upon request from the corresponding authors. General Matlab functions are available at Matlab Central File Exchange #32,398 and #3733.

## References

[1] Woolsey TA, Van der Loos H (1970). The structural organization of layer IV in the somatosensory region (SI) of mouse cerebral cortex. The description of a cortical field composed of discrete cytoarchitectonic units. Brain Res..

[2] Feldmeyer D (2013). Barrel cortex function. Prog. Neurobiol..

[3] Staiger JF, Petersen CCH (2021). Neuronal circuits in barrel cortex for whisker sensory perception. Physiol. Rev..

[4] Narayanan RT, Udvary D, Oberlaender M (2017). Cell type-specific structural organization of the six layers in rat barrel cortex. Front. Neuroanat..

[5] Harris KD, Mrsic-Flogel TD (2013). Cortical connectivity and sensory coding. Nature.

[6] Douglas RJ, Martin KA (2007). The butterfly and the loom. Brain Res. Rev..

[7] Feldmeyer D, Qi G, Emmenegger V, Staiger JF (2018). Inhibitory interneurons and their circuit motifs in the many layers of the barrel cortex. Neuroscience.

[8] Staiger JF, Möck M, Proenneke A, Witte M (2015). What types of neocortical GABAergic neurons do really exist?. e-Neuroforum.

[9] Tremblay R, Lee S, Rudy B (2016). GABAergic interneurons in the neocortex: From cellular properties to circuits. Neuron.

[10] DeFelipe J (2013). New insights into the classification and nomenclature of cortical GABAergic interneurons. Nat. Rev. Neurosci..

[11] Ascoli GA (2008). Petilla terminology: Nomenclature of features of GABAergic interneurons of the cerebral cortex. Nat. Rev. Neurosci.

[12] Griffen TC, Maffei A (2014). GABAergic synapses: Their plasticity and role in sensory cortex. Front. Cell. Neurosci..

[13] Rudy B, Fishell G, Lee S, Hjerling-Leffler J (2011). Three groups of interneurons account for nearly 100% of neocortical GABAergic neurons. Dev. Neurobiol.

[14] Gentet LJ (2012). Functional diversity of supragranular GABAergic neurons in the barrel cortex. Front. Neural Circ..

[15] Helm J, Akgul G, Wollmuth LP (2013). Subgroups of parvalbumin-expressing interneurons in layers 2/3 of the visual cortex. J. Neurophysiol..

[16] Karnani MM, Agetsuma M, Yuste R (2014). A blanket of inhibition: Functional inferences from dense inhibitory connectivity. Curr. Opin. Neurobiol..

[17] Sohal VS, Zhang F, Yizhar O, Deisseroth K (2009). Parvalbumin neurons and gamma rhythms enhance cortical circuit performance. Nature.

[18] Pritchett DL, Siegle JH, Deister CA, Moore CI (2015). For things needing your attention: The role of neocortical gamma in sensory perception. Curr. Opin. Neurobiol..

[19] Cardin JA (2009). Driving fast-spiking cells induces gamma rhythm and controls sensory responses. Nature.

[20] Siegle JH, Pritchett DL, Moore CI (2014). Gamma-range synchronization of fast-spiking interneurons can enhance detection of tactile stimuli. Nat. Neurosci..

[21] Ferguson BR, Gao WJ (2018). PV interneurons: Critical regulators of E/I balance for prefrontal cortex-dependent behavior and psychiatric disorders. Front. Neural Circ..

[22] Yang, J. W., Prouvot, P. H., Stroh, A. & Luhmann, H. J. in *Optogenetics: A Roadmap* (ed A. Stroh) Ch. 8, 133–152 (Springer, 2018).

[23] Pesaran B (2018). Investigating large-scale brain dynamics using field potential recordings: Analysis and interpretation. Nat. Neurosci..

[24] Buzsáki G, Anastassiou CA, Koch C (2012). The origin of extracellular fields and currents - EEG, ECoG, LFP and spikes. Nat. Rev. Neurosci..

[25] Han X (2011). A high-light sensitivity optical neural silencer: Development and application to optogenetic control of non-human primate cortex. Front. Syst. Neurosci..

[26] Yang JW (2017). Optogenetic modulation of a minor fraction of parvalbumin-positive interneurons specifically affects spatiotemporal dynamics of spontaneous and sensory-evoked activity in mouse somatosensory cortex in vivo. Cereb. Cortex.

[27] van der Bourg A (2017). Layer-specific refinement of sensory coding in developing mouse barrel cortex. Cereb. Cortex.

[28] Ewert TA, Vahle-Hinz C, Engel AK (2008). High-frequency whisker vibration is encoded by phase-locked responses of neurons in the rat's barrel cortex. J. Neurosci..

[29] Andermann ML, Moore CI (2006). A somatotopic map of vibrissa motion direction within a barrel column. Nat. Neurosci..

[30] Goldberg JM, Brown PB (1969). Response of binaural neurons of dog superior olivary complex to dichotic tonal stimuli: Some physiological mechanisms of sound localization. J. Neurophysiol..

[31] Lovett-Barron M, Losonczy A (2014). Behavioral consequences of GABAergic neuronal diversity. Curr. Opin. Neurobiol..

[32] Kann O (2016). The interneuron energy hypothesis: Implications for brain disease. Neurobiol. Dis..

[33] Agetsuma M, Hamm JP, Tao K, Fujisawa S, Yuste R (2018). Parvalbumin-positive interneurons regulate neuronal ensembles in visual cortex. Cereb. Cortex.

[34] Neske GT, Patrick SL, Connors BW (2015). Contributions of diverse excitatory and inhibitory neurons to recurrent network activity in cerebral cortex. J. Neurosci..

[35] Zucca S, Pasquale V, Lagomarsino de Leon RP, Panzeri S, Fellin T (2019). Thalamic drive of cortical parvalbumin-positive interneurons during down states in anesthetized mice. Curr. Biol..

[36] Crochet S, Poulet JFA, Kremer Y, Petersen CCH (2011). Synaptic mechanisms underlying sparse coding of active touch. Neuron.

[37] Sachidhanandam S, Sreenivasan V, Kyriakatos A, Kremer Y, Petersen CCH (2013). Membrane potential correlates of sensory perception in mouse barrel cortex. Nat. Neurosci..

[38] Atallah BV, Bruns W, Carandini M, Scanziani M (2012). Parvalbumin-expressing interneurons linearly transform cortical responses to visual stimuli. Neuron.

[39] Fu T (2021). Exploring two-photon optogenetics beyond 1100 nm for specific and effective all-optical physiology. iScience.

[40] Katzner S, Busse L, Carandini M (2011). GABAA inhibition controls response gain in visual cortex. J. Neurosci..

[41] Mitchinson B (2011). Active vibrissal sensing in rodents and marsupials. Philos. Trans. R. Soc. Lond. B Biol. Sci..

[42] Sofroniew NJ, Cohen JD, Lee AK, Svoboda K (2014). Natural whisker-guided behavior by head-fixed mice in tactile virtual reality. J. Neurosci..

[43] Fois C, Prouvot PH, Stroh A (2014). A roadmap to applying optogenetics in neuroscience. Methods Mol. Biol..

[44] Reyes-Puerta V, Sun JJ, Kim S, Kilb W, Luhmann HJ (2015). Laminar and columnar structure of sensory-evoked multineuronal spike sequences in adult rat barrel cortex in vivo. Cereb. Cortex.

[45] Erceg-Hurn DM, Mirosevich VM (2008). Modern robust statistical methods: An easy way to maximize the accuracy and power of your research. Am. Psychol..

[46] Hentschke H, Stüttgen MC (2011). Computation of measures of effect size for neuroscience data sets. Eur. J. Neurosci..

[47] Schneider CA, Rasband WS, Eliceiri KW (2012). NIH Image to ImageJ: 25 years of image analysis. Nat. Methods.

