# Supplementary material for: Effects of optogenetic inhibition of a small fraction of parvalbumin-positive interneurons on the representation of sensory stimuli in mouse barrel cortex
Source: Sci Rep. 2022 Nov 12;12:19419. doi: 10.1038/s41598-022-24156-y (PMC9653449; doi:10.1038/s41598-022-24156-y)
Supplement: Supplementary file 1 — Supplementary Information. [file 41598_2022_24156_MOESM1_ESM.docx]

**Supplementary Information**

**Effects of optogenetic inhibition of a small fraction of parvalbumin-positive interneurons on the representation of sensory stimuli in mouse barrel cortex**

**Fahimeh Yeganeh ^1,2^, Beate Knauer ^2^, Roberta Guimarães Backhaus ^3^,**

**Jenq-Wei Yang ^1^, Albrecht Stroh ^2,3^, Heiko J. Luhmann ^1+^ & Maik C. Stüttgen ^2+^**

^1^ Institute of Physiology, University Medical Center of the Johannes Gutenberg University Mainz, Mainz, Germany

^2^ Institute of Pathophysiology, University Medical Center of the Johannes Gutenberg University Mainz, Mainz, Germany

^3^ Leibniz Institute for Resilience Research, Mainz, Germany

^+^ These authors share senior authorship.

## **Supplemental Figure 1**


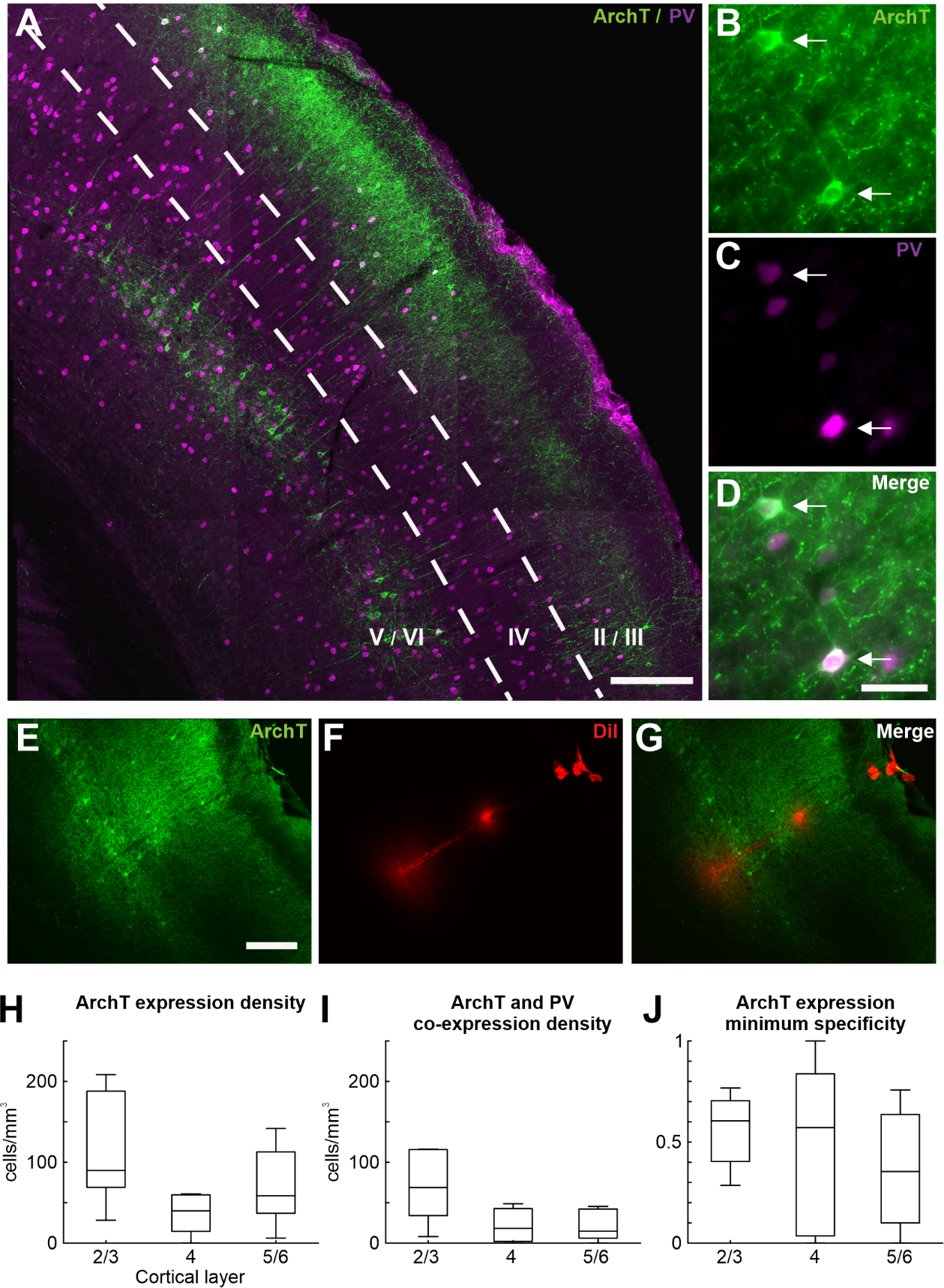


**Suppl. Figure 1: Quantification of opsin-expressing neurons and specificity of opsin expression**. (A) Apotome mosaic micrographs of a coronal slice of the mouse barrel cortex showing the ArchT opsin injection site, ArchT expression (green) and immunohistochemistry of PV expression (magenta). Scale bar 50 µm. (B–D) Cropped region of an Apotome micrograph showing ArchT opsin expression (B, green), immunohistochemistry of PV expression (C, magenta) and merge (D) of both channels. Scale bar 10 µm. The two white arrows show ArchT and PV cells co-expression pattern. (E–G) Apotome micrograph of coronal slice of mouse barrel cortex showing ArchT opsin expression (E, green) and probe penetration site (F, DiI, red) and merge (G). Scale bar 50 µm. (H–I) Boxplots of ArchT (H) and ArchT/PV (I) cell expression density across supragranular, granular, and infragranular layers. J) Specificity of expression across cortical layers (upper whisker 90% CI, upper box boundary 75% CI, median, lower box boundary 25% CI, lower whisker 10% CI).
